# Mitigation of Hepatotoxicity via Boosting Antioxidants and Reducing Oxidative Stress and Inflammation in Carbendazim-Treated Rats Using *Adiantum Capillus-Veneris* L. Extract

**DOI:** 10.3390/molecules28124720

**Published:** 2023-06-12

**Authors:** Mohamed Seif, Hanan Aati, May Amer, Arthur J. Ragauskas, Amr Seif, Ahmed H. El-Sappah, Abdulrahman Aati, Abd El-Nasser A. Madboli, Mahmoud Emam

**Affiliations:** 1 Toxicology and Food Contaminants Department, Food Industries and Nutrition Research Institute, National Research Centre, Dokki, Giza 12622, Egypt; mayamer7@yahoo.com; 2 Pharmacognosy Department, College of Pharmacy, King Saud University, P.O. Box 22452, Riyadh 11495, Saudi Arabia; 3 Department of Chemical and Biomolecular Engineering, University of Tennessee, Knoxville, TN 37996-2200, USA; aragausk@utk.edu; 4 Faculty of Medicine, Assuit University, Asyut 71516, Egypt; amrseif.medic@gmail.com; 5 Genetics Department, Faculty of Agriculture, Zagazig University, Zagazig 44511, Egypt; ahmed_elsappah2006@yahoo.com; 6 Rokn Al-Madaein Pharmaceutical Warehouse Co., P.O. Box 2990, Riyadh 11495, Saudi Arabia; abdulrahman11aati@gmail.com; 7 Animal Reproduction and Artificial Insemination Department, Veterinary Research Institute, National Research Centre, Dokki, Giza 12622, Egypt; abdelnasser_mazen_monzer@yahoo.com; 8 Phytochemistry and Plant Systematics Department, National Research Centre, Dokki, Giza 12622, Egypt; mahmoudemamhegazy2020@gmail.com

**Keywords:** *A. capillus-veneris* L., carbendazim, GC-MS, hepatotoxicity, protection, rats

## Abstract

Exposure to food contaminants continues to be a substantial source of human health risks all over the world, particularly in developing countries. Carbendazim (CBZ) is a chemical fungicide used to control the spread of various fungi and other pathogens in the agriculture and veterinary sectors. The hazardous effects of CBZ on human health occur due to the accumulation of its residues in agricultural food products. In this study, the possible hepatoprotective effects of *Adiantum capillus-veneris* L. (*ACVL*) extract were evaluated in CBZ-treated rats. A GC-MS analysis revealed that *ACVL* extract contained several bioactive hydrocarbon components and fatty acids, and that the components exerted hepatic protection by mitigating oxidative stress via upregulating antioxidant agents and neutralizing nitrogen and oxygen free radicals. Moreover, *ACVL* extracts relieved hepatic inflammation via decreasing NO, NF-κB, and pro-inflammatory cytokines (TNF-a, IL-6) in the liver of CBZ-treated rats, both at protein and mRNA levels. In addition, the protective effect of *ACVL* has appeared in the histopathological figures and function markers in the livers of CBZ-treated rats. According to the present results, *ACVL* extract can protect the hepatic tissue and restore its functions to a control level in CBZ-treated rats; this effect may be attributed to its antioxidant and anti-inflammatory activities.

## 1. Introduction

Although the public standards for pesticide residues have become stricter than ever before, pesticide residues are still one of the major worldwide categories of food contaminants, especially in developing countries. Fungicides are chemical compounds used to prevent the spread of pathogenic fungi in the agriculture and veterinary sectors; they can cause serious spoiling, resulting in the loss of crop yields and thus lessening expected revenues [1]. Despite this, pesticides are still one of the most acceptable and effective means of protecting plants and animals against pest attacks, and have contributed significantly to enhanced agricultural output. However, uncontrolled application of pesticides results in the continuous exposure of humans and non-target organisms to pesticide residues that accumulate in environmental components and reach the food chain, representing a key source of contamination of food and feeds [2].

Carbendazim (methyl-2-benzimidazole carbamate-CBZ) is a broad-spectrum benzimidazole systemic fungicide used to preclude the spread of molds and rots in vast fruits and vegetables [3]. Additionally, CBZ is strongly absorbed into soil, and remains in the soil for up to three years [4]; afterwards, it is again taken up by plants through their roots, seeds, or leaves, before being transferred to the whole plant [5]. Concentrations of CBZ in plants increase with increasing dose rate; thus, the repeated field applications of this fungicide may yield a high accumulation of its residues in the soil, and thence in agricultural products samples [3]. Recent publications have reported that CBZ has been found in serial, fruits, and soil samples in different regions, including Egypt [6,7,8,9]. The liver is a vital organ in the human body; it is responsible for many important functions, such as xenobiotic detoxification, and is the organ most sensitive to pesticide poisonousness [10]. Based on recent research, exposure to some carbamate pesticides, including CBZ, can induce hepatic lipid peroxidation, inflammation, and oxidative stress [11,12].

Oxidative stress is an impairment of the equilibrium between reactive oxygen species (ROS) and antioxidants on behalf of reactive oxidants. ROS act by causing pathological changes in the cellular membrane, cellular organelles, and DNAs through the oxidation of proteins, lipids, and carbohydrates. As a result of this, functional impairment or cell death may develop, and tumors may develop by gaining mutant features [13]. Glutathione is the most abundant tripeptide; it plays an important role in maintaining cellular redox and protecting the cells against oxidative stress [14].

It is known that several products of medicinal plants have promising therapeutic and pharmacological activities; they can exert their effects by enhancing antioxidant defense systems in experimental animals and can protect bodily organs against many environmental and food contaminants [2,15,16,17,18]. *Adiantum capillus-veneris* L. (*ACVL*) is a cosmopolitan species widely distributed in tropical climates and areas of high humidity. *ACVL* is one such plant, reputed to have numerous useful applications in traditional medicine.

In brief, previous phytochemical reports have demonstrated the existence of different carotenoids such as pheophytin derivatives, carotene epoxide, xanthin derivatives, neochrome, lutein, chlorophyll a, and b; terpenes such as isoadiantone, isoadiantol-B, 3-methoxy-4-hydroxyfilican,e and 3,4 dihydroxyfilicane; phenolic acid derivatives such as 4-hydroxybenzoic acid, *O*-caffeoylquinic acid, 2-caffeoyl tartaric acid, p-Coumaric acid, rosmarinic acid, gentisic acid, coumaric acid derivative, and ferulic acid; flavonoids and their derivatives, such as kaemferol-3-feruloylsophoroside-7-glucoside, quercetin hexoside, caffeic acid hexoside, kaempferol-3-*O*-sophorotrioside, quercetin rhamnoside-hexoside, quercetin-3-galactoside, apigenin-7-*O*-glucoside, isorhamnetin-3-*O*-di-glucoside, epicatechin 7-*O*-rutinoside, and kaempferol 3-*O*-glucoside; quercetin-3-*O*-glucoside, quercetin-3-*O*-rutinoside (rutin), and Kaempferol 3-sulphat [19,20,21,22,23]; and sulphate esters of hydroxycinnamic acid sugars [19]. 

Many published reports have demonstrated that *ACVL* was used as an antitussive and anti-fever treatment and as a treatment for digestive disorders [24]. Moreover, many studies have reported its antimicrobial [25,26], anti-inflammatory [27], and antioxidant activities [28]. In this regard, the present study aims to evaluate the non-polar profile of *ACVL* ethanolic extract using GC/M and to evaluate its ability to protect the rat liver, following exposure to CBZ, by boosting the antioxidant system.

## 2. Results

### 2.1. Phytochemical Analysis

Phytochemical evaluation of *ACVL* extracts demonstrated the good antioxidant capability of the *ACVL* extract against ATPS and DPPH radicals, as well as the extract containing a high amount of TPC and TFC (Table 1).

#### GC-MS Analysis

Based on the previously published LC-MS, HPLC, and phytochemical results, which indicated the presence of compounds terpenes, flavonoids, phenolics, and sulfated compounds [19,20,21,22,23,29] in the *ACVL* extract, in the current study, we only focused on the non-polar profile of the extract that had not been completely investigated before.

**Figure 1:**
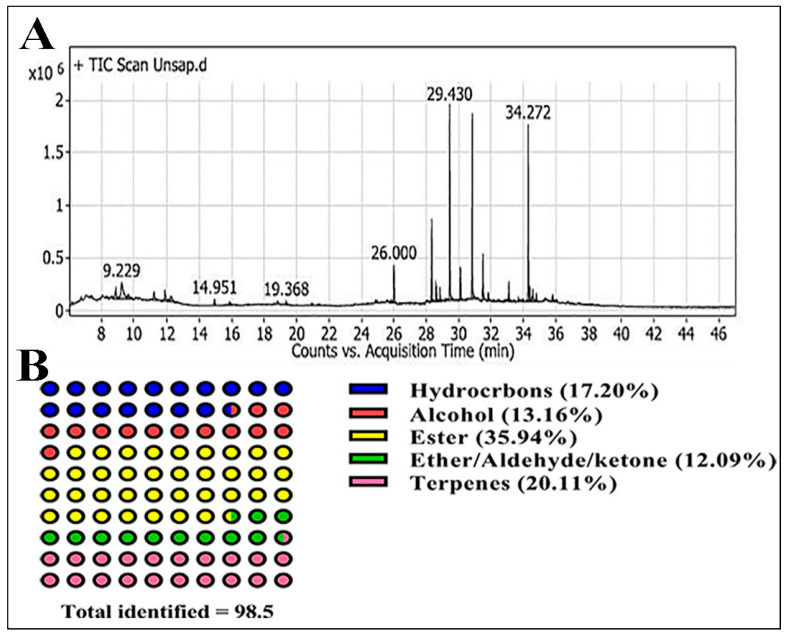
Total ion chromatogram (TIC) of GC-MS of the unsaponifiable matter of *ACVL* (**A**); illustration of the percentage of different classes (**B**).

The hydrocarbon and fatty acid contents were identified using GC-MS. The data illustrated in Figure 1 and Table 2 revealed that the UNSAP extract of *ACVL* contains 31 components, classified as terpenes (20.11%), hydrocarbons (17.2%), esters (35.94%), oxygenated compounds (25.25%) of alcohols, ethers, aldehyde, and ketone structures.

The palmitoyl–glycerol structure showed the most predominant fraction, with 16.14%, while linoleoyl–glycerol represented the lowest, with 0.24%. In addition, the campesterol structure was determined as the major steroidal structure, with 15.36%, while the pentacyclic triterpene of betulin represented 0.77%, as the lowest terpene structure.

**Figure 2:**
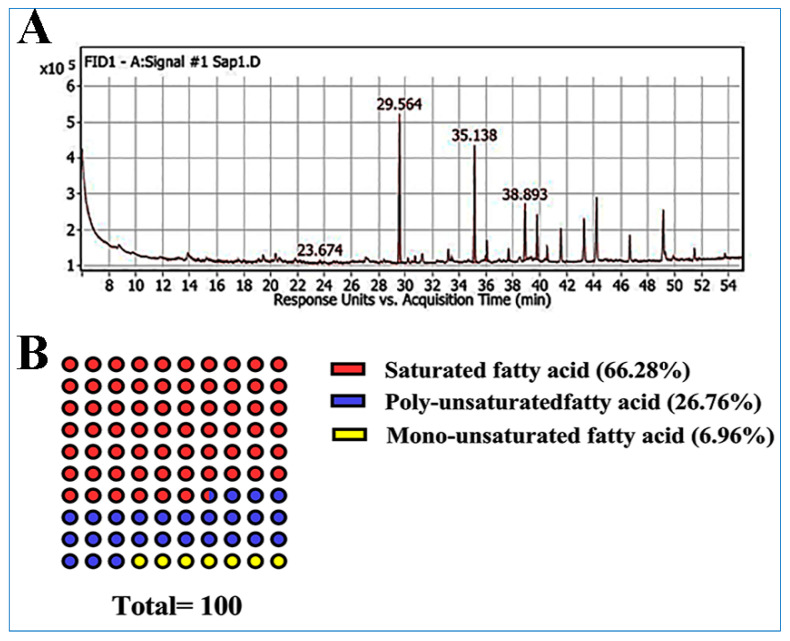
Total ion chromatogram (TIC) of GC-MS of the fatty acids of *ACVL* (**A**); illustration of the percentage of different classes of fatty acids (**B**).

Moreover, the data illustrated in Figure 2 and Table 3 showed that the saponifiable matter (SAP) extract of *ACVL* contains ten fatty acids, which are descendingly ordered as palmitic acid, stearic acid, γ-linolenic acid, linolenic acid, oleic acid, linoleic acid, arachidic acid, palmitoleic acid, cis-10-heptadecenoic acid, and myristic acid, respectively, according to their peak areas. The saturated fatty acids composed 66.28%, while the unsaturated fatty acids represented 33.72%. The most prominent saturated fatty acid was palmitic acid, with 35.91%, while γ-Linolenic acid was the major unsaturated fatty acid, with 13.8%. 

### 2.2. General Clinical Symptoms, Body Weight, and Liver Weight

**Figure 3:**
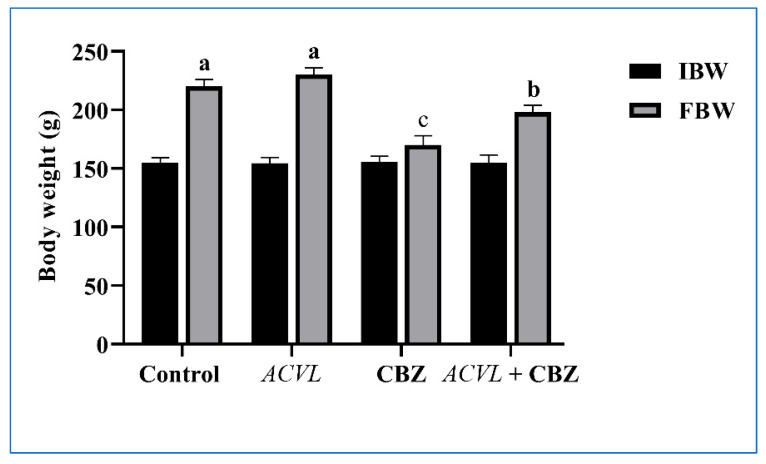
Change in body weight of rats treated with *Adiantum Capillus-Veneris* L. (*ACVL*) and carbendazim (CBZ) at the end of the experiment. The different letters represent statistically significant differences (*p* < 0.05) between treatment and control.

No mortality cases were recorded in all experimental rats during the treatment period. Rats treated with CBZ showed varieties of clinical signs such as weakness, slight hair loss, and a reduction in appetite. Rats in groups treated with *ACVL* extract either alone or simultaneously with CBZ exhibited normal performance and good health. The data in Figure 3 illustrate the effects of CBZ, and *ACVL* on body weight and liver weight. The body weight of animals treated with CBZ for 30 days dwindled significantly; however, the liver weight increased compared to that of the control rats. Meanwhile, the supplementation of *ACVL* extracts led to significant improvements in body weight and liver weight compared with rats treated with CBZ (*p* ≤ 0.05). In the same regard, treatment with *ACVL* extract alone did not cause any significant changes in liver weight.

### 2.3. Histopathological Findings

**Figure 4:**
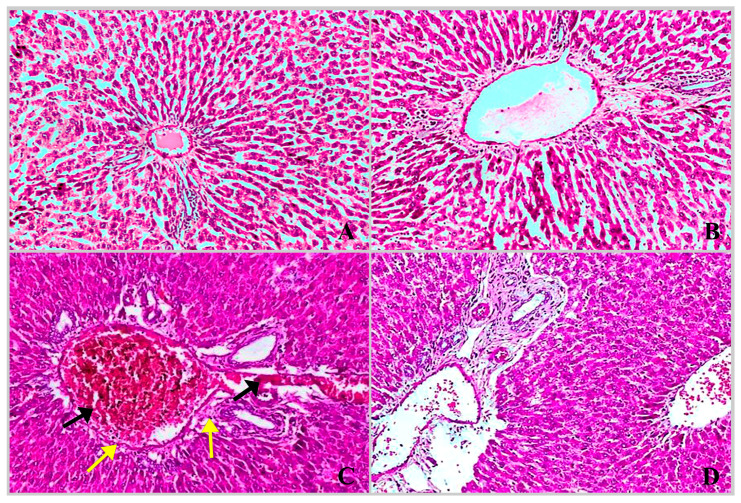
Liver section photomicrographs of rats in the control group and *ACVL*-treated rats ((**A**,**B**), respectively) exhibited normal histological structures of hepatic tissues without any pathological changes (×100). A photomicrograph of a liver section of CBZ-treated rats (**C**) displayed severe dilatation and congestion in the portal vein branch and blood sinusoids (black arrows-×100). Moreover, the CBZ led to focal and periportal necrosis (yellow arrow) of the hepatocytes that surrounded the portal area (yellow arrows). The liver section of *ACVL* + CBZ-treated rats (**D**) revealed recovery and an absence of congestion and hemorrhage in the hepato-portal area (×100).

The hepatic and renal tissues of the experimental rat groups were examined histopathologically and showed the following findings: the liver of the control rats (Figure 4A) and *ACVL*-treated rats (Figure 4A,B) showed normal liver hepatocytes and portal veins, with no histopathological changes. The slide of the liver of CBZ-treated rats clearly illustrated that severe dilatation and congestion were found in the portal vein branch and blood sinusoids; this is associated with mild dilatation in the bile duct (Figure 4C). Furthermore, the slide of the liver of *ACVL* + CBZ-treated rats showed that congestion and hemorrhage in hepato-portal circulation are nearly eliminated compared to the slide of CBZ-treated rats.

### 2.4. Oxidative Stress and Antioxidant Biomarkers

**Figure 5:**
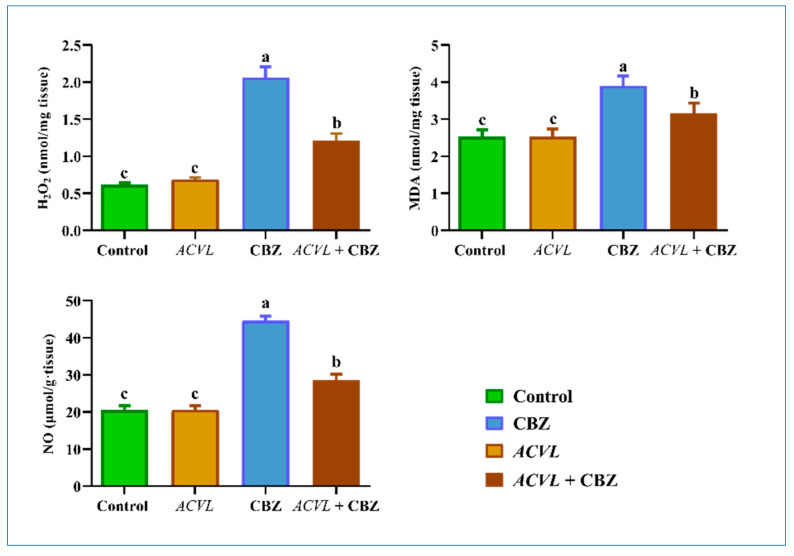
Hepatic oxidative stress biomarkers in different experimental groups. Data arerepresented as mean ± SD (n = 7). *ACVL* = *Adiantum Capillus-Veneris* L. CBZ = carbendazim; H_2_O_2_ = hydrogen peroxide; MDA = malondialdehyde. The different letters represent statistically significant differences (*p* < 0.05) between treatment and control.

Malondialdehyde (MDA) and hydrogen peroxide (H_2_O_2_) showed an extremely significant boost in CBZ-treated rats compared with the control group. Meanwhile, the co-administration of *ACVL* extract led to a significant alleviation in MDA and H_2_O_2_ compared to their levels in the CBZ-treated rats (Figure 5; *p* ≤ 0.05).

**Figure 6:**
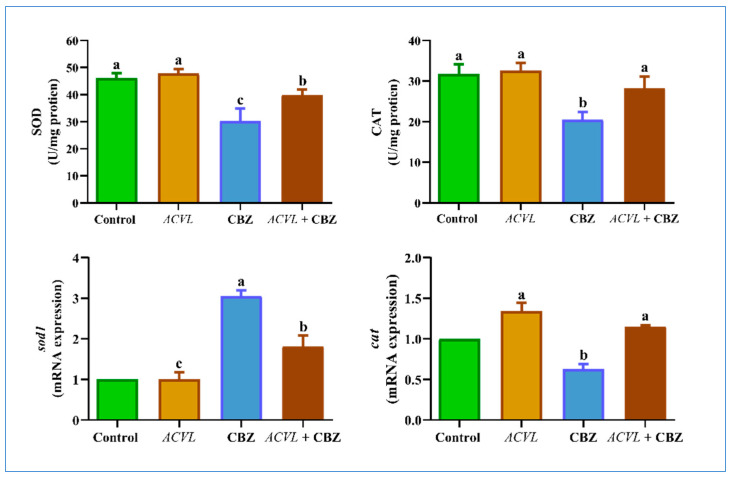
Hepatic antioxidant biomarkers at protein and gene levels in different experimental groups. Data are represented as mean ± SD (n = 7). *ACVL* = *Adiantum Capillus-Veneris* L. CBZ = carbendazim; SOD = superoxide; dismutase; CAT = catalase. The different letters represent statistically significant differences (*p* < 0.05) between treatment and control.

Moreover, the current findings demonstrated that levels of the antioxidant enzymes superoxide dismutase and catalase (SOD and CAT) and their responsible genes were markedly diminished in the CBZ-treated rats compared with the untreated group. However, concurrent treatment with *ACVL* extract plus CBZ markedly enhanced the levels of endogenous antioxidant enzymes (SOD and CAT) and their genes when compared with CBZ-treated only. (Figure 6). The results proved that the supplementation of *ACVL* extract is safe and did not cause any changes in oxidative stress and endogenous antioxidant biomarkers when compared with the untreated group.

### 2.5. GSH-Related Antioxidant Biomarkers

**Figure 7:**
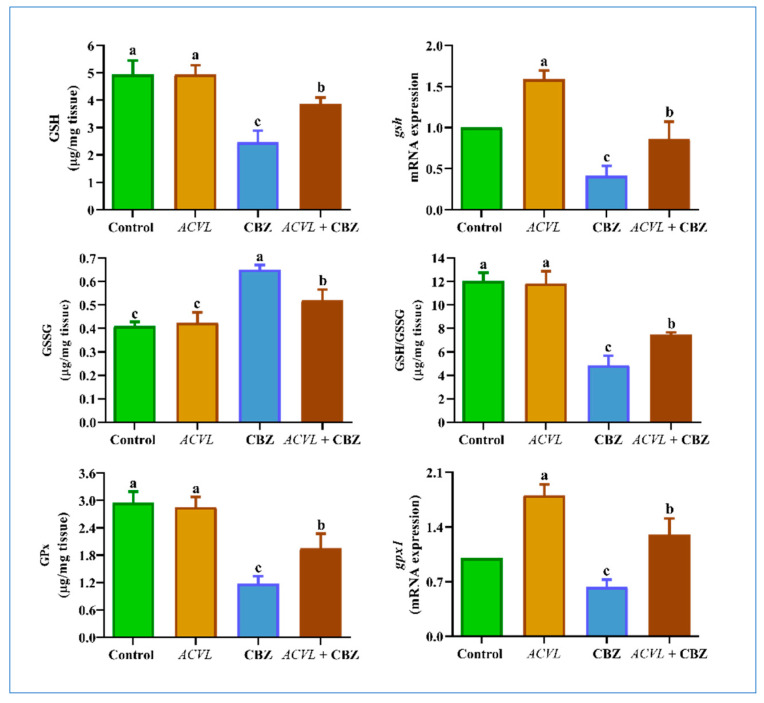
Hepatic glutathione system-related biomarkers at protein and gene levels in different experimental groups. Data are represented as mean ± SD (n = 7). *ACVL* = *Adiantum Capillus-Veneris* L. CBZ = carbendazim; GSH = glutathione; GSSG = GSH disulfide; GPx = glutathione peroxidase; GSH/GSSG = the ratio between reduced glutathione and glutathione disulfide. The different letters represent statistically significant differences (*p* < 0.05) between treatment and control.

A momentous reduction in GSH-related biomarker (GSH, Gpx, GSH/GSSG ratio) levels was observed in the liver homogenate of CBZ-treated rats (*p* < 0.05). However, concurrent treatment with *ACVL* and CBZ considerably restored the GSH content to its original level in the CBZ-treated rats. The oxidizing glutathione form (GSSG) was significantly raised in CBZ-treated rats compared to the control rats. The *ACVL* extract significantly improved the status of the level of GSSG and the GSH/GSSG ratio, therefore nullifying the negative effects observed in CBZ-treated rats (Figure 5). The same trend shown by these results has been observed in gpx1 and *gsh* genes (Figure 7).

### 2.6. Serum Hepatic Function Biomarkers

**Figure 8:**
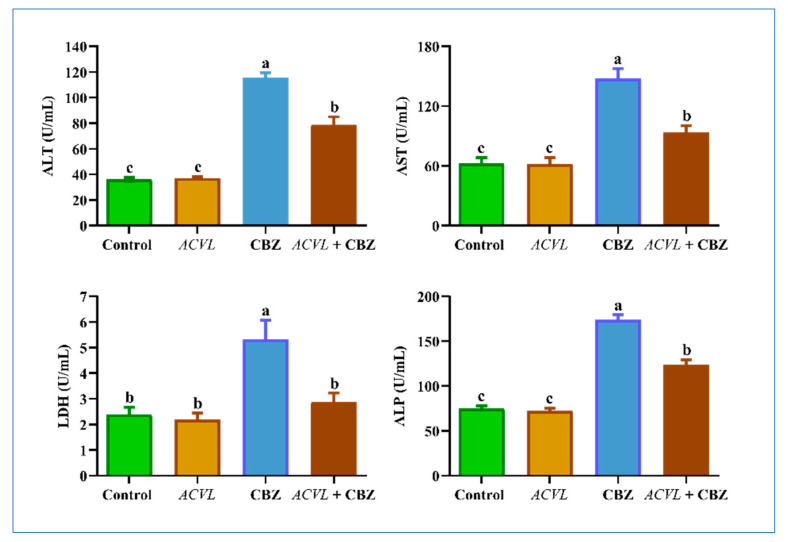
Biomarkers of liver injury in different experimental groups. Data represented as mean ± SD (n = 6). ALT = alanine amino transaminase, AST = aspartate aminotransaminase, ALP alkaline phosphatase, LDH = lactate dehydrogenase; *ACVL* = *Adiantum Capillus-Veneris* L. CBZ = carbendazim. The different letters represent the statistically significant differences (*p* < 0.05) between treatment and control.

Compared to control rats, CBZ-treated rats showed a noticeable increase in the serum levels of their hepatic function markers (ALT, AST, ALP, and LDH). Concurrent treatment with *ACVL* + CBZ caused a significant ameliorative effect on the serum liver function, compared to those in the CBZ group alone. In addition, the results demonstrated that *ACVL* treatment alone did not show a significant change in liver function compared to the control group (Figure 8).

### 2.7. Hepatic Inflammation Biomarkers

The protein level of NF-κB, TNF-α, and IL-6 was found to be curiously higher in the CBZ group compared to the control group, and the concomitant treatment with *ACVL* and CBZ restored NF-κB, TNF-α, and IL-6 levels compared to their levels in CBZ-treated rats. Furthermore, the same effects of CBZ have been noted in the hepatic mRNA levels of NF-κB, TNF-α, and IL-6, which were significantly elevated under CBZ treatment compared to their levels in the control group. However, when CBZ-treated rats received *ACVL* extract, they displayed an absolute improvement in the mRNA levels compared with those in CBZ-treated rats, which emphasizes the influential role of the *ACVL* in mitigating cellular complications related to CBZ toxicity.

**Figure 9:**
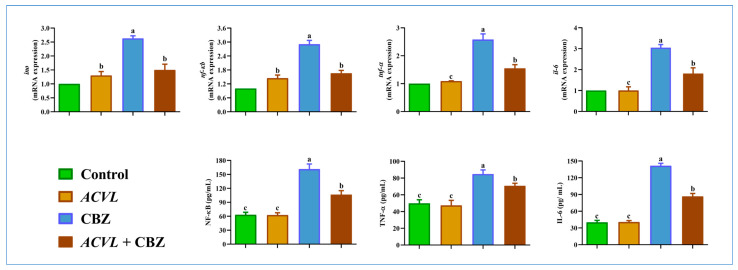
Hepatic inflammation biomarkers of NF-κB, TNF-α, and IL-6, of protein and gene expression in different experimental groups. Data are represented as mean ± SD (n = 6). NF-κB = NF-kappa B; TNFα = tumor necrosis factor-alpha; IL6 = interleukin; inos = inducible nitric oxide; *ACVL* = *Adiantum Capillus-Veneris* L. CBZ = carbendazim. The different letters represent statistically significant differences (*p* < 0.05) between treatment and control.

Outstandingly, the effect of *ACVL* was found to be comparable in the control group. Sequentially, CBZ exposure induced liver inflammation in the treated rats; this was evidenced by a significant trigger of NF-κB generation, which can induce the pro-inflammatory biomarkers (TNF-α, and IL-6) to increase in comparison with their levels in the control group (Figure 9).

### 2.8. Detection of the NF-κB (IHC)

**Figure 10:**
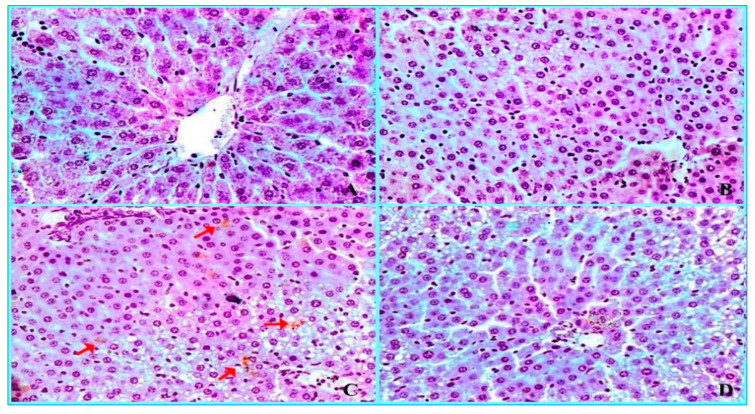
Immunohistochemistry photomicrographs of detection of NF-κB in the different treated groups. The slides of control and *ACVL*-treated groups (**A**,**B**) showed a negative immunoreaction response against NF-κB-P65 in the liver tissues. Meanwhile, the slide of the CBZ-treated group (**C**) showed positive brown-stained immunoreaction bands toward the NF-κB-P65 antigen (red arrows). The slide of the group treated with both *ACVL* + CBZ showed no immunoreaction response toward the NF-κB-P65 antigen (**D**).

We carried out the detection of the NF-κB-P65 protein, an inflammatory marker in the tissues of the female reproductive system, stomach, spleen, and brain. The examined tissues were stained with di-amino-benzidine stain (DAB), as a vital stain, and hematoxylin stain as a background. According to IHC photomicrograph slides, the control and *ACVL*-treated groups (A and B) showed negative immunoreaction responses to NF-κB-P65 in the liver tissues. The slide of the CBZ-treated group (C) showed positive brown-stained immunoreaction bands toward the NF-κB-P65 antigen. Moreover, the slide of the group treated with both *ACVL* + CBZ showed no immunoreaction response toward the NF-κB-P65 antigen (Figure 10).

## 3. Discussion

Liver injury involving characteristic reactive oxygen/nitrogen species (RONS) and inflammation plays a key role in the progression of liver disease [30]. Pesticide residues are one of the most perilous food/feed contaminants, which usually reach the food chain due to uncontrolled pesticide applications either in the field or in storage, causing negative effects on human and animal health. Free radicals are continuously produced in the body, as a fundamental mediator in completing various normal biochemical processes in the body. However, exposure to pesticide residues may lead to an extreme amount of reactive oxygen/nitrogen species (RONS) being engendered, which alters the antioxidant defense mechanism and triggers oxidative stress and inflammation [30]. Physiologically, the body’s cells have many ways in which they can alleviate the deleterious effects of oxidative stress, either directly diminishing the harmful effects of oxidative stress by employing enzymatic and non-enzymatic antioxidants, or by repairing the incurred damage [31].

Antioxidants are compounds that usually prevent lipid and protein oxidation sequence oxidative stress-related diseases, including inflammation and even cancer, in the organism body [32]. These include phenolics, vitamins, fatty acids, dietary flavonoids, and some natural active components that are isolated from natural products [18,32]. However, amounts of these protective devices present under normal physiological conditions are sufficient only to cope with the normal threshold of a physiological rate of free radical generation. Otherwise, any additional burden of free radicals, either from an indigenous or exogenous source, leads to oxidative imbalance, itself leading to oxidative stress and afterward contributing to the development of serious pathological conditions [33].

Nowadays, significant attention is being paid to herbal therapy scenarios, through applying phytochemicals and herbal extracts as food additives and promising sustainable agent alternatives to chemotherapeutic drugs to treat the harmful effects of environmental and food contaminants. In addition, herbal extracts are more accessible, have fewer side effects, and are cost-effective, compared to chemotherapeutic agents [34]. In this regard, the present study planned to investigate the protective ability of *ACVL* extract to counteract the hepatotoxic effects of carbendazim in rats by studying its ability to improve the antioxidant performances of enzymatic (superoxide dismutase and catalase) and non-enzymatic antioxidants (glutathione). 

The current results of the phytochemical analysis of the *ACVL* extract show that it had a good scavenging ability against free radicals (DPPH and ABTS), and showed a high content of TPC and TFC; these results concur with those reported by Nasrollahi et al. [35] and Roy et al. [36]. Moreover, the GC/MS analysis showed that the extract contained 32 hydrocarbons and 10 fatty acids, components assumed to exert antioxidant effects. 

Furthermore, the current findings revealed that there was a significant decrease in the body weights of rats treated with CBZ pesticide, accompanied by a significant increase in liver weight. The significant differences in the body weight of CBZ-treated rats and the control rats reflect the deleterious effects of CBZ. Similar observations were conveyed in previous studies [37,38]. A lesser appetite could be responsible for the reduced body weight gain in CBZ-treated groups. This may be also indicative of CBZ-induced toxicity hampering the basal metabolism of the body [39]. The current result showed that the decrease in body weight following exposure to CBZ pesticide may be attributed to its ability to induce oxidative stress, which leads to a decreased appetite, which will result in a direct decrease in body weight loss.

These results are confirmed by the histopathological findings, which showed that many pathological signs were noted in the hepatic tissues of rats treated only with CBZ. These results corresponded with those published by Mo et al. [40], and Abolaji et al. [41]. Concomitant treatment with *ACVL* and CBZ relieved the histopathological events in the hepatic tissues; this effect is attributed to the antioxidative capabilities of the *ACVL* extract, which combat the oxidative stress and tissue damages. These results are in line with the results of Nasrollahi, Talebi and Bashardoost [35].

The study of biochemical parameters could help to identify target organs of toxicity and the general health status of the experimental animals. Moreover, it may also provide an early warning signal in the stressed organism. The disruption of the level of any parameter is indicative of a response to environmental effects, and can also serve as a marker for toxicant exposure [42].

The liver plays a major role in the detoxification of xenobiotics, protein synthesis, the regulation of cell functioning, etc. Disturbed hepatic redox homeostasis under oxidative stress is sufficient to alter normal physiological function [43].

Our findings suggest that CBZ-induced oxidative stress in hepatic tissues increases the level of (H_2_O_2_) and (MDA). However, the results showed a strong decline in the level of SOD, CAT, Gpx, and GSH, and their RNA levels, which led to disruption of normal homeostasis and induced oxidative stress in hepatic tissues in the treated rats. These results were in accordance with the findings of Owumi et al. [44], and Patil et al. [45].

NO, lipid peroxidation, and antioxidant enzymes are used as parameters of oxidative stress, an alteration commonly found in natural and experimental models [46,47]. The cytotoxicity of NO may be due to its ability to produce peroxynitrite, starting a variety of oxidative responses, including alterations of nucleic acids, lipids, and proteins that cause tissue grievance [48]. Chronic increases in NO, besides oxidative events in cells, are a key biochemical event that has been linked to cancer and inflammation.

NF-κB is a transcription factor that regulates the expression of numerous genes, such as cytokine genes, that elaborate in immune and inflammatory responses. The disruption of cellular homeostasis leads to the activation of NF-κB via proteolytically degraded IκB. As a result of NF-κB’s activation, it will translocate into the nucleus for the induction of its proinflammatory cytokines [49,50].

The enzymatic antioxidant defense system mainly includes SOD, CAT, and Gpx; these enzymes work together as harmonized systems to protect cells against oxidative stress by neutralizing the excessive reactive oxygen/nitrogen species (RONS). The SOD converts the superoxide anion radicals (O^2−^) to hydrogen peroxide (H_2_O_2_), and CAT splits the generated hydrogen peroxide into water and oxygen [51,52]. Even though Gpx is the most abundant selenoprotein in mammals, selenoenzyme spurs the oxidation of GSH to GSSG, and thereby scours the H_2_O_2_ [53].

Glutathione is a key antioxidant defense in the organism’s body; it acts either by reacting and scavenging several free reactive species or by catalyzing numerous antioxidant enzymes such as Gpx and GST [54]. Hepatic GSH is maintained at a constant concentration through GSH synthesis and turnover. GSH is derived from three amino acids (cysteine, glutamate, and glycine); GSH synthesis occurs in the cytosol through two ATP-consuming steps. The overall sinusoidal efflux of GSH is electrogenic and thiol- and disulfide-sensitive. Uncharged thiols such as dithiothreitol (DTT) stimulate the efflux, while disulfides such as cysteine and glutathione disulfide (GSSG) decrease the efflux [55]. In addition, extracellular methionine trans-inhibits the efflux of GSH, while the hepatocytes export GSH into the plasma to maintain inter-organ GSH homeostasis [56].

The present results disclosed that treatment with *ACVL* treatment in CBZ-treated rats caused a significant improvement in GSH, SOD, CAT, and GPx activity, and their RNA levels in the liver homogenated toward their activities in CBZ rats. Additionally, synchronous treatment with *ACVL* extract enhanced the content of GSH and decreased the oxidizing form of GSSG, compared with its content in rats treated with CBZ alone. This may be attributed to the antioxidant capabilities of *ACVL*, which have been proven using phytochemical evaluation results, and agrees with those results issued by [57,58]. Moreover, *ACVL* treatment enhances the activities of GSH, as an antioxidant promoting the protection of live tissues against the toxic effects of CBZ via combated oxidative stress.

*ACVL* has already been applied to treat liver tumors and hepatic disorders [59,60]. The activity of antioxidant enzymes, maintained at a normal level using co-administration of *ACVL*, indicates the potent role of *ACVL* as an antioxidant. These protection roles of *ACVL* extract may be related to its ability to limit oxidative stress and DNA damage [61,62]. These observations assert the hypothesis that the production of oxidative damage is one of the principal mechanisms implicated in CBZ toxicity, and that *ACVL* extract plays a pivotal role in the prevention of oxidative damage induced by CBZ. 

Additionally, our results indicate that inflammation markers (NO, NF-κB, IL-6, and TNFα) are significantly raised in tissues of rats treated with CBZ; here, we refer to NF-κB, which is activated and induce the production of inflammatory mediators following CBZ treatment in experimental rats. These observations have also been noted in the level of RNA of *ino*, *nf-κb*, *il-6*, and *tnfα*, proving the inflammatory effects of CBZ in treated rats. 

Inflammation is a common defense mechanism against many stressors and pathogenic diseases such as microbial and viral infections, radiation and toxic chemicals, autoimmune and chronic diseases, and hazardous food behaviors [63]. The relationship between oxidative stress and inflammation has been documented by many authors. Scientific evidence has indicated that oxidative stress plays a pathogenic role in the induction of chronic inflammatory diseases [64]. Under stressors, excessive ROS was produced, which led to the induction of inflammation; in the current study, CBZ caused significant generation of free radicals. Otherwise, per the current findings, treatment with *ACVL* mitigated the inflammatory response in the liver of CBZ-treated rats, and the levels of NO, NF-κB, IL-6, and TNFα were restored to the control level. These observations are in agreement with those issued by [65]. In addition, according to Yuan et al., [66] the ethanolic extract of *A. capillus-veneris* L. had an anti-inflammatory effect through inhibiting NF-κB activation and suppressing the production of inflammatory mediators such as NO, IL-6, and TNFα.

According to the current observations, the hepatic oxidative stress and inflammation caused by CBZ treatment acted to disrupt the liver function; this was evident in the significant elevation in liver function biomarkers (ALT, AST, ALP, and LDH). These findings are in accordance with those results published by Sharma et al. [67], and with the elevation of AST, ALT, and ALP, which are considered important biomarkers for hepatic damage. Indeed, AST and ALT are two hepatic enzymes that will be released in the blood in the event of cellular destruction, in a manner proportional to the intensity of cellular aggression [68]. Moreover, our results indicated that a high positive parallel between serum rates of AST, ALT, and hepatic MDA levels is consistent with the damage to the hepatic tissues in CBZ-treated rats, as seen using light microscopy. When the liver cell membrane is damaged, several enzymes located in the hepatocyte cytosol, including ALT, AST, ALP, and LDH, are secreted into the blood [69]. Consequently, these enzymes are a biomarker of liver damage [70]. Similarly, a high level of serum LDH is an indicator of hepatic necrosis [68].

Moreover, co-treatment using *ACVL* and CBZ restored the liver functions to a control level. The protective effects of *ACVL* extract have been documented previously [71,72]. Importantly, supplementation with *ACVL* extract led to significant protection of the liver tissues from lipid oxidative stress and inflammation responses, and improved hepatic function signs. This effect might be attributed to its antioxidants, and anti-inflammation activities [65,73]. The antioxidant and anti-inflammatory effects of *ACVL* were attributed to its bioactive contents. The GC/MS findings showed that the extract is enriched by numerous bioactive components that can act as free radical scavengers, antioxidants, and anti-inflammatory agents. Moreover, according to the GC/MS data, the SAP fraction of the *ACVL* extract enriched with ten fatty acids, palmitic acid, stearic acid, gamma-linolenic acid, linolenic acid, and oleic acid, was found to be a major fraction of the extract. 

Many published reports have shown that saturated fatty acids have many health-beneficial activities [74], such as antioxidant [75,76,77] and anti-inflammatory effects [78,79]. According to current results, ten fatty acids were identified in *ACVL* extract; palmitic acid, stearic acid, linolenic acid, and oleic acid were found in high concentrations, according to their peak areas. These fatty acids exert antioxidant and anti-inflammatory effects.

## 4. Materials and Methods

### 4.1. Chemicals and Kits

Carbendazim (97% pure), was obtained from Sigma (St. Louis, MO, USA), and high-purity-grade ethanol was purchased from Merck (Darmstadt, Germany). All chemicals used in this study were HPLC grade. The alanine amino-transaminase (ALT), aspartate amino-transaminase (AST), alkaline phosphatase (ALP), hydrogen peroxides (H_2_O_2_), superoxide dismutase (SOD), catalase (CAT), and nitric oxide (NO) were obtained from BIODIAGNOSTICS Co. (Cairo, Egypt). 

### 4.2. Plant Materials and Extract Preparation

The fresh plants were collected from some farms in the Monufiya governorate of Egypt. Aerial parts were cleaned and dried, and one hundred grams were extracted by soaking in 1 L (50% ethanol) and then heated for one hour. Then, the extract was filtrated through Whatman no. 1 filter paper. The solvent was fully evaporated from the residues using a rotary evaporator [27] at 35 °C. The extract was freeze-dried and stored at −4 °C. The freeze-dried powder extract was dissolved in corn oil, and then the dose concentration was adjusted according to the weight of the experimental rats.

### 4.3. Phytochemical Analysis

#### 4.3.1. Quantitative Estimation of Phenolic and Flavonoid Contents

The total phenolic content of *ACVL* extract was determined with Folin–Ciocalteau reagent (FCR), using gallic acid as standard, and expressed as milligrams of gallic acid equivalents (GAE)/g dry extract [80]. After incubation for 2 h at 25 °C, the mixture was measured at 765 nm using a Shimadzu UV-visible spectrophotometer (1800 UV-probe). The content of total flavonoids was calorimetrically determined using an aluminum chloride assay based on the quercetin calibration curve and expressed in milligrams of quercetin equivalent per milligram of dry extract (QE). The absorbance was measured at 415 nm [81]. Estimates were conducted in triplicate.

#### 4.3.2. Evaluation of the Radical Scavenging Abilities of *ACVL*

##### ABTS Assay

To perform the ABTS assay, ABTS·+ cation radical was generated by incubating the mixture of 7.0 mM ABTS and 2.45 mM potassium persulfate at 25 °C in darkness for 12–14 h. ABTS·+ solution was then diluted with methanol to obtain an absorbance of 0.700 at 734 nm. Then, 5 μL of *ACVL* extract was added to 4 mL of diluted ABTS·+ solution. The absorbance was detected exactly 30 min from initial mixing. All the measurements for the samples and blank were performed at least three times, and the finding values were expressed as milligrams of Trolox equivalents per g of dry extract (mg TE/g DE).

##### DPPH Free Radical Scavenging Assay

The free radical scavenging potential of the *ACVL* extracts was determined according to the procedure conveyed by [82], with insignificant modifications. In brief, 1 mL of *ACVL* extract was mixed with 3 mL of methanolic DPPH solution (0.004%) and stored the mixture in the dark for 30 min. Then, the absorbance of the mixture was measured at 517 nm spectrophotometrically (UV-Vis 3000, ORI, Berlin, Germany). All the measurements for the samples and blank were performed as replicates. The ascorbic acid was used as the standard, with methanol as the negative control. The percentage inhibition was evaluated using the following equation. The results were assumed as IC_50_. % inhibition = 100 × (AC − AS)/AC, where AC = absorption of the control sample, and AS = absorption of the test sample.

#### 4.3.3. Identified the Hydrocarbon and Fatty Acid Contents by GC/MS

GC/MS was used to detect the hydrocarbon and fatty acid contents in the SAP and UNSAP fractions. Ethanolic *ACVL* extract was saponified (SAP) with ethanolic potassium hydroxide, and the unsaponifiable (UNSAP) fraction was extracted in petroleum ether. The sample of the saponified fraction was derivatized using the added mixture of 50 µL of bis-trimethylsilyl trifluoroacetamide (BSTFA), trimethylchlorosilane (TMCS) 99:1 sialylation reagent, and 50 µL pyridine, before GC/MS analysis.

##### Gas Chromatography-Mass Spectrometry Analysis (GC/MS)

The GC-MS system (Agilent Technologies) was equipped with a gas chromatograph (7890B) and mass spectrometer detector (5977A) at Central Laboratories Network, National Research Centre, Cairo, Egypt. The GC was equipped with an HP-5MS column (30 m × 0.25 mm internal diameter and 0.25 μm film thickness). Analyses were carried out using Hydrogen as the carrier gas at a flow rate of 2.0 mL/min at a splitless, injection volume of 2 µL, and the following temperature program: 50 °C for 5 min, rising at 5 °C/min to 100 °C and held for 0 min, and rising at 10 °C/min to 320 °C and held for 10 min. The injector and detector were held at 280 °C, and 320 °C, respectively. Mass spectra were obtained using electron ionization (EI) at 70 eV, using a spectral range of *m*/*z* 25–700 and solvent delay of 6 min. The mass temperature was 230 °C and Quad 150 °C. Identification of different constituents was carried out by comparing the spectrum fragmentation patterns with those stored in Wiley and NIST Mass Spectral Library data.

##### Gas Chromatography for Fatty Acids Methyl Ester (FAME)

The GC model 7890B from Agilent Technologies was equipped with a flame ionization detector at the Central Laboratories Network, National Research Centre, Cairo, Egypt. Separation was achieved using a Zebron ZB-FAME column (60 m × 0.25 mm internal diameter × 0.25 μm film thickness). Analyses were carried out using hydrogen as the carrier gas at a flow rate of 1.8 mL/min and in a split 1:50 mode. An injection volume of 1 µL was used, alongside the following temperature program: 100 °C for 3 min; rising at 2.5 °C/min to 240 °C and held for 10 min. The injector and detector (FID) were held at 250 °C and 285 °C, respectively.

### 4.4. Biological Evaluation of ACVL Extract

#### 4.4.1. Animals and Treatments

**Figure 11:**
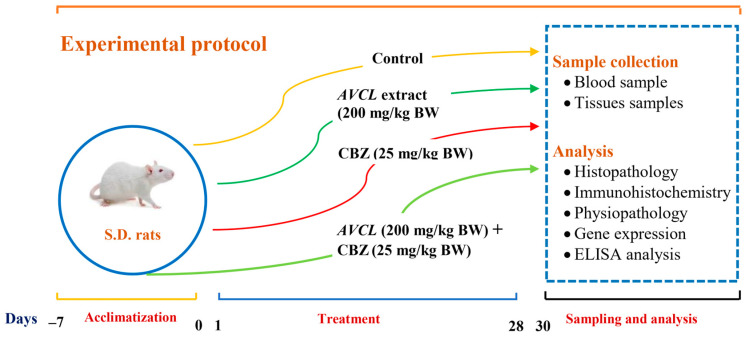
Schematic of biological experiments. *ACVL* = *Adiantum Capillus-Veneris* L.; CBZ = Carbendazim; S.D. rats = Sprague Dawley rats; ELISA = enzyme-linked immunosorbent assay.

In the present study, forty (32) Sprague Dawley rats were provided by an animal house colony, National Research Centre, Cairo, Egypt, weighing 150 ± 5 g. All the animals were acclimatized to laboratory conditions for seven days before treatment; animals were fed a commercial pellet diet and provided with water ad libitum. The CBZ and *ACVL* extract dosage was given in normal saline as follows. The experimental line and animal handling were managed following experiments approved by the Committee of Animal Ethics in the National Research Centre, Dokki, Cairo, Egypt (No: 19062). Animals were divided randomly into four groups (n = 8) and fed a standard basal diet, dosed once daily through the oral route, for 28 days, sequentially, as follows and described in Figure 11.

- The control group: animals served as controls and were given normal saline.
- The *ACVL* group: animals were treated orally with plant extract (200 mg kg^−1^ BW).
- The CBZ group: animals were treated orally with CBZ (25 mg kg^−1^ BW), according to [27,83].
- The *ACVL*+ CBZ group: animals were given *ACVL* extract (200 mg kg^−1^ BW) and CBZ (25 mg kg^−1^ BW) orally.

During treatment, clinical symptoms, mortality, and body weight were recorded daily. On day 30, the blood samples were collected for biochemical analysis; then, the rats were sacrificed via cervical dislocation. Liver tissues were dissected out and washed immediately with ice-cold physiological saline (0.9% NaCl); other portions of the liver tissues were fixed in formalin solutions for histopathological investigation.

#### 4.4.2. Histopathological Inspection

The liver tissues were removed immediately after decapitation and fixed in formalin solution (10%), then dehydrated in an ascending series of alcohol, cleared in two changes of xylene, and embedded in paraffin wax. Sections of 4–5 μm thickness were cut using a rotary microtome and mounted on clean slides. For histopathological examination, sections were stained with Ehrlich’s hematoxylin and eosin [84].

#### 4.4.3. Oxidative Stress Markers

##### Preparation of Liver Tissue Homogenates

The liver tissues were homogenized in 0.9% NaCl, using an Ultra Turrax tissue homogenizer to make up the 10% homogenate (*w*/*v*), then centrifuged at 7500 rpm at 4 °C for 10 min to obtain the cytosolic fraction. The (10%) homogenates were used to determine the oxidative stress and inflammation biomarkers.

##### Oxidative Stress Biomarkers

The level of hydrogen peroxide (H_2_O_2_) was determined as described by Pick and Keisari [85]. Briefly, the assay is based on the horseradish peroxidase-mediated oxidation of phenol red using H_2_O_2_. The reaction mixture contained 0.28 mmol phenol red, 8.5 units of horseradish peroxidase, and 0.2 mL tissue homogenate. After 5 min of incubation, 1mL of 0.1 N NaOH was added, and the tube was read at 610 nm. The results were expressed in nmoles of H_2_O_2_ per gram of tissue. Malondialdehyde (MDA) was assayed spectrophotometrically via a reaction with thiobarbituric acid [86]. NO measured as nitrite was determined in tissue homogenates using Griess reagent. Nitrate was converted to nitrite via nitrate reductase. Subsequently, the nitrite was converted with Griess reagent to a deep purple azo compound, which can be detected using a spectrophotometer [87].

#### 4.4.4. Hepatic Functions Biomarkers

Sera samples were obtained via centrifugation of the blood samples, and stored at −20 °C before assessment for the biochemical parameters. Aspartate aminotransferase (AST) and alanine aminotransferase (ALT) were measured calorimetrically according to Reitman [88]. Alkaline phosphatase (ALP) was measured according to the method of Belfield and Goldberg [89] using the kit supplied by Biodiagnostic, Egypt. Moreover, the level of lactate dehydrogenase (LDH) was measured based on a method published by Wang et al. [90] using the LDH obtained from Jian Cheng Bioengineering Institute, Nanjing, China.

#### 4.4.5. Antioxidant Enzymatic Markers

The SOD activity was estimated in liver homogenates using the method of Nishikimi et al. [91] at 440 nm, using a kit purchased from Biodiagnostic, Cairo, Egypt. The activity of CAT was assayed in the liver and kidney homogenates using decomposition of hydrogen peroxide (H_2_O_2_), according to the protocol of Aebi [92], in the 10% liver homogenates.

#### 4.4.6. GSH System Markers

The hepatic activities of GSH, GSSG, and GPx were determined using commercial kits obtained from Nanjing Jiancheng Bioengineering Institute (Nanjing, China). All measurements were meticulously performed in line with the instructions of the manufacturer.

#### 4.4.7. Determination NF-κB, TNF-α, and IL-6 in Liver Tissue

The NF-κB, TNF-α, and IL-6 levels in the liver tissue were determined using ELISA kits purchased from Nanjing Jiancheng Bioengineering Institute (Nanjing, China). All measurements were performed following the manufacturer’s guidelines.

#### 4.4.8. Detection of the NF-κB (IHC)

The IHC protocol was applied to the paraffinized blocks of liver tissue, first sectioned at 3 µm and stuck on positive charge slides. Antigen retrieval of NF-κB P65 protein was performed by dipping the slides into sodium citrate buffer 10% as a retrieval solution, then autoclaving for 5 min at 20–25 PSI pressure (pounds per square inch). Blocking of the nonspecific binding was performed on the tissue slides using the super-blocking reagent that came with the kit (before the specific primary antibody was used). The tissue slides were incubated with rabbit anti-NF-κB p65 IgG2a, diluted in PBS buffer of pH 7.4 with a rate of dilution of 1:100, containing 0.9% sodium azide (NaN_3_) and 0.2% bovine serum albumin (BSA) [93]. A CRFTM anti-polyvalent polymer stain peroxidase detection kit, imported from Scy-Tek Laboratories, USA, was used. The species specificity of the kit is anti-rabbit and anti-mouse. The control negative for the tested tissue was incubated with normal saline instead of the primary antibody. Additionally, a control positive tissue specimen (rat stomach tissue that had previously shown strong positive results for NF-κB p65 via IHC) was inserted into the test. Tissue slides were counterstained with hematoxylin and examined under a light microscope. The positive findings range from light brown and golden brown to deep brown, according to the antigenic intensity in the examined tissues in comparison to the positive and negative control slides [94].

#### 4.4.9. qPCR Analysis

The expression level of antioxidant and inflammation-related genes was evaluated in liver tissues. Briefly, total RNA was isolated from the 20 mg of liver tissue using Trizol reagent (Invitrogen, Waltham, MA, USA) following the manufacturer’s procedures. After evaluating the concentration and solidity of extracted RNA, the RNA was treated using RNase-free DNase I (Promega, Madison, WI, USA), and then reversed to complementary DNA (cDNA) according to the manufacturer’s instructions. The conversion into cDNA was carried out using SuperScript Reverse Transcriptase kits (Thermo Fisher Scientific, Waltham, MA, USA). The expression levels of manganese-dependent superoxide dismutase (*Sod1*), catalase (*Cat*), glutathione peroxidase (*Gpx1*), nuclear factor kappa B (*NF-κB*), tumor necrosis factor-alpha (*Tnfa*), and interleukin-6 (*IL-6*) assessed in the liver tissue using real-time quantitative reverse transcription polymerase chain reaction (qRT-PCR) with an Applied Biosystems 7500 Instrument. Relative gene expression was determined using the 2^−ΔΔCT^ technique from three replicates. Glyceraldehyde 3-phosphate dehydrogenase (*Gapdh*) was used as a housekeeping gene [95]. The primer sequences used are listed in Table 4.

### 4.5. Statistical Analysis

Data are presented as mean ± SD, and a *p*-value < 0.05 was considered statistically significant. A statistical test was performed using (SPSS, 11.5) software. We performed a one-way analysis of variance followed by Duncan’s multiple range test (DMRT) for multiple comparisons between the groups. GraphPad Prism-8 was used to create the figures.

## 5. Conclusions

Worldwide, especially in developing nations, exposure to the chemical fungicide carbendazim (CBZ) as a food contaminant continues to be a significant source of danger to human health. Additionally, medicinal plant extracts have promising therapeutic and pharmacological effects that can be used to improve antioxidant defense mechanisms against numerous environmental and dietary toxins. To summarize, the observations herein revealed that *ACVL* extract mitigated CBZ toxicity in hepatic tissues of CBZ-treated rats, because it relieved the inflammation, combated the oxidative stress, upregulated the antioxidant genes, downregulated the NF-κB and pro-inflammatory genes, relieved the negative pathological signs, and improved hepatic functioning following CBZ exposure. The results showed that *AVCL* extract has admirable antioxidant and anti-inflammatory activities against the toxicity of pesticide residues.

## Figures and Tables

**Table 1:** TPC, TFC, ABTS, and DPPH values for *Adiantum Capillus-Veneris* L. extract.

	TPC (mg GAE/g Extract)	TFC (mg QE/g Extract)	ABTS (mg TE/g Extract)	DPPH (IC_50_ µg/mL)
*ACVL* extract	86.16 ± 0.41	74.24 ± 0.37	58.52 ± 0.8	92.05 ± 0.42
Ascorbic acid	-	-	-	30.08 ± 0.02

*ACVL* = *Adiantum Capillus-Veneris* L.; TPC = total phenolic components; Total Flavonoids components; ABTS = 2,2-azinobis 3-ethylbenzothiazoline-6- sulfonic acid; DPPH = 2,2-diphenyl-2-picrylhydrazyl; IC_50_ = half maximal inhibitory concentration; mg = milligram; g = gram.

**Table 2:** Chemical compositions of unsaponifiable matter (UNSAP) of *Adiantum Capillus-Veneris* L. as identified using GC-MS analysis.

No.	Rt (min)	rRT	Metabolites	Molecular Formula	Area Sum %	Class
1	8.87	0.30	4-ethenyl-3,8-Dioxatricyclo [5.1.0.0(2,4)] octane	C_8_H_10_O_2_	2.76	Diepoxides
2	9.22	0.31	Hexadecanal	C_16_H_32_O	7.46	Aldehydes
3	9.62	0.32	2,4-Hexadien-1-ol	C_6_H_10_O	1.67	Alcohols
4	11.22	0.38	6-methyl-Octadecane	C_19_H_4_0	1.78	Hydrocarbons
5	11.89	0.40	Pentadecan-8-ol	C_15_H_32_O	1.89	HC alcohol
6	12.24	0.42	Valeric acid, 2-pentadecyl ester	C_20_H_40_O_2_	1.9	Ester
7	14.95	0.51	Glycerol	C_3_H_8_O_3_	0.73	Alcohol
8	15.89	0.54	2-methoxy-2-methyl-Propane	C_5_H_12_O	0.46	Ether
9	19.36	0.66	(E)-5-Tetradecen-3-yne	C_14_H_24_	0.42	Hydrocarbons
10	20.95	0.71	Bicyclo [2.2.0] hex-1-yl-methanol	C_7_H_12_O	0.22	Bicyclic alcohol
11	24.86	0.85	5β,7βH,10α-Eudesm-11-en-1α-ol	C_15_H_26_O	0.77	Monoterpene hydrocarbons
12	25.84	0.88	Phytol	C_20_H_40_O	0.67	Acyclic diterpene alcohol
13	26	0.88	2-tert-Butyl-4-methylphenol	C_11_H_16_O	3.94	Phenylpropanes
14	28.32	0.96	1-chloro-Octadecane	C_18_H_37_Cl	6.4	Hydrocarbons
15	28.60	0.97	Undec-10-ynoic acid, dodecyl ester	C_23_H_42_O_2_	1.8	Ester
16	28.83	0.98	4-(3-tert-butyl-4-hydroxy-5-methylbenzyl)-2-tert-butyl-6-methylphenol	C_23_H_34_O_2_	1.16	Phenol
17	29.19	0.99	Undec-10-ynoic acid, dodecyl ester	C_23_H_42_O_2_	0.29	Ester
18	29.43	1	1-Monopalmitin	C_19_H_38_O_4_	16.14	Ester
19	29.63	1.01	1b,5,5,6a-Tetramethyl-octahydro-1-oxa-cyclopropa[a]inden-6-one	C_13_H_20_O_2_	0.49	Epoxy/keto
20	30.09	1.02	Heptacosane	C_27_H_56_	3.29	Hydrocarbons
21	30.81	1.05	2,3-dihydroxypropyl stearate	C_21_H_42_O_4_	15.57	Ester
22	31.47	1.07	Docosane	C_22_H_46_	4.54	Hydrocarbons
23	31.81	1.08	(Z, Z)-9,12-Octadecadienoyl chloride	C_18_H_31_ClO	0.92	Ketone
24	32.13	1.09	γ-Tocopherol	C_28_H_48_O_2_	0.25	Chroman-6-ol
25	33.07	1.12	1-Octacosanol	C_28_H_58_O	2.25	fatty alcohol
26	33.67	1.14	12-Methyl-E, E-2,13-octadecadienoic-1-ol	C_19_H_36_O	0.38	fatty alcohol
27	34.27	1.16	Campesterol	C_28_H_48_O	15.36	Sterols
28	34.37	1.17	4-methyl-, (3β,4α)-Cholesta-8,24-dien-3-ol	C_28_H_46_O	1.79	Sterol
29	34.55	1.17	Stigmasterol	C_29_H_48_O	2.19	Sterol
30	34.77	1.18	Betulin	C_30_H_50_O_2_	0.77	Pentacyclic triterpene
31	36.01	1.22	1-Monolinoleoylglycerol	C_21_H_38_O_4_	0.24	Ester

Relative percentage (%) of total identified compounds 98.5%, rRT: retention time relative to that of 1-Monopalmitin (Rt = 29.43 min).

**Table 3:** Chemical compositions of fatty acids of *Adiantum Capillus-Veneris* L. identified using GC-MS analysis.

Peak	RT	rRT	Name	Area %	Class
1	23.6	0.80	Myristic acid	0.57	Saturated fatty acid
2	29.5	1	Palmitic acid	35.91	Saturated fatty acid
3	30.7	1.03	Palmitoleic acid	1.42	Monounsaturated fatty acid
4	33.4	1.13	*cis*-10-Heptadecenoic acid	1.21	Monounsaturated fatty acid
5	35.1	1.18	Stearic acid	27.32	Saturated fatty acid
6	36.0	1.21	Oleic acid	4.33	Monounsaturated fatty acid
7	37.6	1.27	Linoleic acid	2.74	Polyunsaturated fatty acid
8	38.8	1.31	γ-Linolenic acid	13.8	Polyunsaturated fatty acid
9	39.7	1.34	Linolenic acid	10.22	Polyunsaturated fatty acid
10	40.5	1.37	Arachidic acid	2.48	Saturated fatty acid

Relative (%) of total identified compounds 100%, rRT: retention time relative to palmitic acid (Rt = 29.56 min).

**Table 4:** Sequences of primer pairs used in the real-time quantitative PCR reactions.

Gene Description	Target Gene	Accession No.	Sequences (5′—3′)
Cu/Zn Superoxide dismutase	*SOD1*	NM_017050.1	F: CATTCCATCATTGGCCGTACT
			R: CCACCTTTGCCCAAGTCATC
Catalase	*CAT*	NM_012520.2	F: GTACAGGCCGGCTCTCACA
			R: ACCCGTGCTTTACAGGTTAGCT
Glutathione	*GSH*	NM_053906.1	F: GGAAGTCAACGGGAAGAAGTTCACTG
			R: CAATGTAACCGGCACCCACAATAAC
Glutathione peroxidase	*GPx1*	NM_030826.4	F: GCGCTGGTCTCGTCCATT
			R: TGGTGAAACCGCCTTTCTTT
Nuclear factor-kappa B	*NF-κB*	NM_01276711.1	F: AATTGCCCCGGCAT
			R: TCCCGTAACCGCGTA
Inducible Nitric Oxide	*iNOS*	NM_012611.3	F: CACCACCCTCCTTGTTCAAC
			R: CAATCCACAACTCGCTCCAA
Tumor necrosis factor-α	*TNF-α*	NM_012675.3	F: GATCGGTCCCAACAAGGAGG
			R: GCTTGGTGGTTTGCTACGAC
Interleukin-*6*	*IL-6*	NM_012589.2	F: AAGCCAGAGTCATTCAGAGCAA
			R: GGTCCTTAGCCACTCCTTCT
Glyceraldehyde3-phosphate dehydrogenase	*GAPDH*	NM_001394060.1	F: CCACCAACTGCTTAGCCCCC
			R: GCAGTGATGGCATGGACTGTGG

## Data Availability

Not applicable.

## Sample Availability

Samples of the compounds are available from the authors.
